# Application of CPBL combined with mind mapping in acute abdomen clinical training to improve post-training competence

**DOI:** 10.3389/fmed.2025.1565531

**Published:** 2025-11-14

**Authors:** Zhou-wei Xu, Wei-dong Chen, Kai-qiang Wang, Tian-le Zhao, Yu-chen Xu, Xing-yu Wang

**Affiliations:** 1 Department of Emergency Surgery, The First Affiliated Hospital of Anhui Medical University, Hefei, Anhui, China; 2 Department of Clinical Medical, The Second Clinical Medical College of Anhui Medical University, Hefei, Anhui, China

**Keywords:** acute abdomen, CPBL teaching, mind mapping, Mini-CEX, DOPS

## Abstract

**Objective:**

This thesis explores the application model and impact of CPBL combined with mind mapping teaching method on the job competency of trainees during internships of acute abdomen.

**Methodology:**

A total of 216 interns rotating through the emergency surgery department at Anhui Medical University’s First Affiliated Hospital from April 2023 to September 2024 were selected as research subjects. They were randomly divided into a CPBL combined with mind mapping teaching group and a traditional lecture-based learning teaching group. During the rotation, they completed Mini-CEX, DOPS, teaching compliance, and satisfaction assessments.

**Results:**

There was no significant difference in general information between the control group and the observation group. Both groups of interns demonstrated good cooperation in their respective teaching activities and made significant progress. The observation group scored higher in patient reception and clinical skills operations, and reported higher satisfaction with the teaching.

**Conclusion:**

Receiving CPBL combined with mind mapping teaching is more effective in enhancing the job competency of interns in the treatment of acute abdomen compared to traditional lecture-based learning teaching, demonstrating valuable potential for promotion in clinical teaching.

## 1 Introduction

Clinical internship constitutes an integral part of medical education, marking the transition for medical students from theoretical learning to clinical practice. It is a critical teaching stage for developing job competency in professional skills, team collaboration and communication abilities, medical ethics, and research capability (1, 2). As an important clinical teaching base, the emergency surgery department of the First Affiliated Hospital of Anhui Medical University has made internship teaching a significant component alongside medical care, research, and undergraduate teaching. Thus, integrating scientific and effective teaching methods focused on prevalent cases of acute abdominal pain, and establishing a reasonable and fair assessment system, is crucial for enhancing the job competency of interns in emergency diagnosis and treatment (3–5). This approach also serves to stimulate the enthusiasm of students undertaking internships in emergency surgery.

Acute abdomen involves the intersection of multiple disciplines such as surgery, internal medicine and gynecology. As a major type of disease in the emergency surgery department, recognized for its complex etiology, rapid onset and progression, severe symptoms, and potential for misdiagnosis, often accompanied by gastrointestinal dysfunction and general discomfort symptoms (6). Therefore, in the teaching process of acute abdomen, it is necessary to guide the interns to integrate interdisciplinary knowledge, accurately analyze the dynamic evolution characteristics of the disease, complete the in-depth collection of medical history and select reasonable auxiliary examinations, so as to cultivate the correct clinical diagnosis and treatment thinking of acute abdomen. Meanwhile, some interns lack the enthusiasm and initiative for internships due to the traditional lecture-style teaching, which further increases the difficulty with clinical teaching of acute abdomen. Insufficient training will directly result in trainees lacking the job competence to diagnose and treat acute abdomen in their future clinical work (7, 8). Case and Problem Based Learning (CPBL), also known as the Clinical Case Teaching Method, employs real acute abdomen cases to present pertinent questions, guiding students to independently research, engage in group discussions, propose solutions, and track therapeutic outcomes, culminating in a summarized educational model (9–12). As an extension of PBL, CPBL maintains a student-centered, case-led, and problem-based approach, addressing any misleading development of standard cases in PBL (13). By integrating symptom-based mind mapping with foundational theories and essential skills in diagnosing and treating acute abdomen, this method enhances students’ comprehensive understanding of the condition. Selecting cases relevant to improving job competency encourages students to think, analyze, and discuss, fostering proactive clinical learning and meeting the modern medical education requirements for emergency surgery internships.

Between April 2023 and September 2024, 216 interns rotating through the First Affiliated Hospital’s emergency surgery department were engaged. Although there is evidence that CPBL and mind mapping teaching play an important role in medical education, the combined application of these two teaching methods has not yet been investigated in the context of acute abdomen training in emergency surgery rotation. Therefore, the CPBL combined with symptom-based mind mapping teaching method was introduced into acute abdomen internship training. Mini-Clinical Evaluation Exercise (Mini-CEX) and Direct Observation of Procedural Skills (DOPS) were used as formative assessment tools, along with evaluations of teaching compliance and satisfaction (14, 15). The assessment indicates significant efficacy in enhancing medical students’ job competency, providing a basis for future reforms in emergency surgery clinical internship education. Findings are reported as follows.

## 2 Materials and methodology

### 2.1 Participants

The study involved 216 interns engaged in emergency surgery teaching, consisting of 120 males and 96 females, aged 20–25 years, with a mean age of (22.48 ± 2.56) years; 185 held an undergraduate degree, and 31 associate degree. Participants were randomly assigned to observation and control groups by a 2:1 ratio (144 and 72) using a random data table method. Initial theory test results confirmed no significant difference in baseline data between groups (Table 1). Inclusion criteria: (1) informed consent provided for this study; (2) completed at least a 2-weeks rotation in emergency surgery; (3) no prior CPBL and mind mapping training; (4) passing entrance assessment score. Exclusion criteria: (1) simultaneous participation in other research; (2) less than a 2-weeks rotation or withdrawal midway; (3) inability to complete clinical teaching training; (4) failing entrance assessment score.

**TABLE 1:** Comparison of the members of the two groups in general information.

Group	Number of cases	Gender		Age (year, $\bar{\text{x}}$± *s*)	Education		Entry grades (score, $\bar{\text{x}}$± *s*)
		Male	Female		Bachelor	College	
Observation group	144	79	65	22.34 ± 2.45	123	21	78.73 ± 5.78
Control group	72	41	31	22.67 ± 2.73	62	10	80.24 ± 7.16
x^2^/*t*		1.625		0.706	0.510		1.374
*P*		0.131		0.592	0.714		0.168

### 2.2 Faculty team

Teaching faculty for intern doctors are selected by the Department of Emergency Medicine with specific criteria: intermediate or higher professional titles, master’s degree or above, a minimum of 5 years of experience, relevant credentials in acute abdomen clinical diagnosis, a teaching qualification or completion of a clinical instructor training course, and completion of CPBL, mind mapping, Mini-CEX, and DOPS training. Instructors should possess comprehensive clinical knowledge to provide appropriate educational training for interns and complete exit assessments objectively and fairly.

### 2.3 Methodology

216 intern doctors participating in the educational research were randomly divided into control and observation groups. The control group engaged only in traditional lecture-based learning teaching, while the observation group received CPBL combined with symptom-based mind mapping instruction, all assessors were blinded to the group allocation of the interns. Each cohort’s learning and assessment period extended for a minimum of 2 weeks. The Mini-CEX and DOPS assessments were scored by three non-instructor staff with intermediate or higher titles from the emergency surgery department, focusing on comparing group performance and progress. Before exiting, interns completed a teaching compliance and satisfaction survey. The above research contents have been approved by the Clinical Medical Research Ethics Committee of the First Affiliated Hospital of Anhui Medical University (Quick-PJ2022-04-42), and all participants provided written informed consent prior to their inclusion in the study.

### 2.4 CPBL teaching

Faculty conduct acute abdomen clinical training in group settings and identify CPBL cases suitable for enhancing job competency. Groups collaboratively review literature using problem-based learning and clinical reasoning, organizing case materials for faculty review. Four to five questions concerning acute abdomen diagnosis, treatment, surgical management, and doctor-patient relations are developed by faculty. Group members create mind maps and discuss these questions; a representative presents and discusses findings inter-group. A summative evaluation by the instructor follows. Interns may also engage in scenario simulations of typical cases, role-playing to deepen their understanding of acute abdomen while enhancing practical skills, with post-simulation improvements based on feedback of teachers (16).

### 2.5 Symptom-based mind mapping instruction

In the observation group, intern doctors use X-Mind software to create mind maps for acute abdomen, with faculty emphasizing mapping principles: (1) A core theme of abdominal pain should be central; (2) Secondary issues arranged succinctly and radially; (3) Use of varied color images for visual impact; (4) Maps should demonstrate comprehensive, easily remembered concepts (17). Faculty select in-patient acute abdomen cases considering job competency improvement requirements while avoiding overly complex or unclear cases to ease mind map creation. By connecting learned points through lateral thinking, students use maps to bridge diseases and form a network. Students are encouraged to discuss during the drawing process to cultivate team spirit, and actively ask the teacher questions to avoid discussion deviation (18).

### 2.6 Evaluation of teaching effectiveness

Intern doctors participating in this educational research undergo assessments, Mini-CEX and DOPS, at department entry and exit, respectively. Mini-CEX focuses on evaluating trainees’ capabilities in handling patient consultations, while DOPS primarily assesses clinical procedural skills (19, 20). The Mini-CEX assessment form, tailored to the clinical attributes of acute abdomen, covers seven aspects: professionalism, communication skills, interviewing skills, clinical judgment, physical examination, procedural skills, and overall evaluation, scored on a 3-tier 9-point scale, with 1–3 points indicating below standard, 4–6 as satisfactory, and 7–9 as excellent. Trainees’ performance in acute abdomen clinical activities is assessed using the DOPS evaluation form, sampling from standard surgical procedures like nasogastric intubation, dressing changes, suture removal, catheterization, and abdominal paracentesis. This evaluation uses an internationally recognized 4-tier 6-point scale, where 1–2 points indicate below standard, 3 as minimally satisfactory, 4 as satisfactory, and 5–6 as excellent. Cooperation of trainees with the teaching model is categorized from low to high as non-compliant, partially compliant, minimally compliant, and fully compliant, serving as a key indicator of the final educational effectiveness. A post-rotation survey assesses student satisfaction with the teaching, focusing on aspects like acceptance of the teaching model, enhancement of clinical reasoning, deepening of knowledge comprehension, and stimulation of learning interest, rated on a 0–10 point scale based on personal experience.

### 2.7 Statistical analysis

The software SPSS 27.0 is used to analyze data collected during this educational research. Quantitative data is expressed as $\bar{x}$ *s*, with inter-group comparisons using the *t*-test; categorical data is expressed as case number or percentage, with inter-group comparisons using the *x*^2^ test. Statistical significance is determined by *P* < 0.05.

## 3 Results

### 3.1 Integration of CPBL teaching and mind mapping for acute abdomen

**FIGURE 1:**
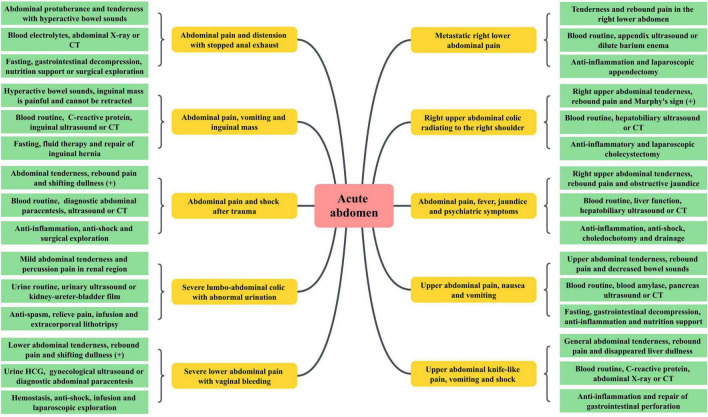
Application of symptom-based mind mapping in diagnosing and treating acute abdomen patients.

**FIGURE 2:**
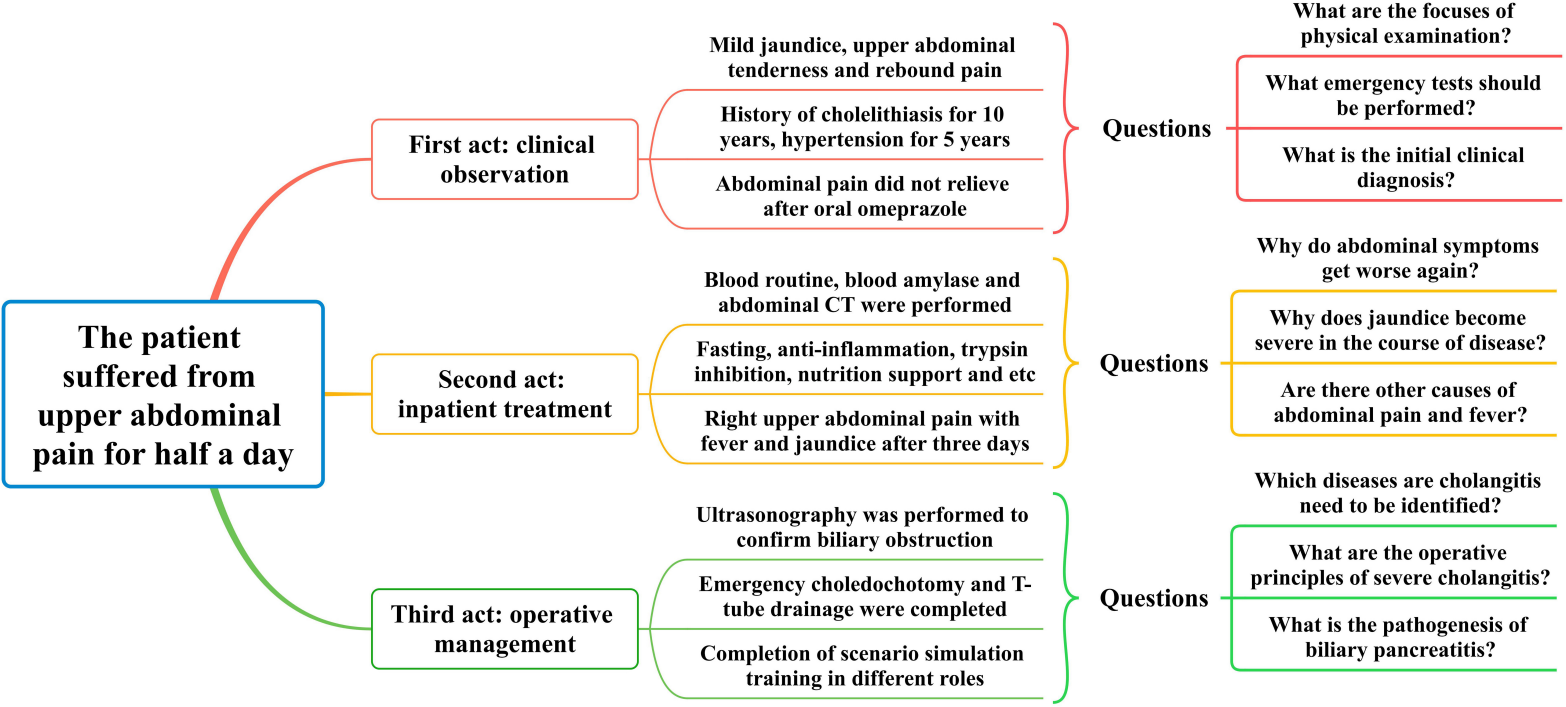
Integration of CPBL with mind mapping in clinical cases of acute abdomen.

Intern doctors in the observation group, after acquiring training related to acute abdomen knowledge, are able to participate effectively in diagnosis and treatment processes. A thorough understanding is achieved regarding patient symptoms, differential diagnosis, treatment principles, surgical decisions, and rehabilitation care. Under faculty guidance, students select clinical acute abdomen cases suitable for CPBL teaching, then generally master the application of X-Mind software and principles for creating mind maps. Team collaboration enables group members to discover, analyze, and resolve case issues, summarizing the entire consultation process for acute abdomen patients based on symptoms in the mind map (Figure 1). The integration of CPBL case analysis with mind mapping renders the diagnosis and treatment of acute abdomen a coherent cognitive process. Furthermore, actively engaging intern doctors in scenario simulations can significantly enhance their job competency, exemplified by the case of a patient with acute abdominal pain illustrated in Figure 2.

Case Overview: The patient is a 58-years-old male who experienced persistent upper abdominal distension and pain after consuming a small amount of alcohol at noon, accompanied by back tightness, nausea, and vomiting of gastric contents. There was no fever, chills, palpitations, or chest tightness. The patient took omeprazole capsules for a presumed gastric issue but saw no improvement and then sought consultation at the hospital. The patient has a 10-years history of cholelithiasis, a 5-years history of hypertension with irregular nifedipine extended-release tablet use, and denies any history of diabetes, coronary artery disease, or surgery. Physical examination revealed a temperature of 37.6 °C, pulse of 110 bpm, and blood pressure of 152/95 mmHg. The skin and sclera were mildly jaundiced, and vesecular breathing sounds were slightly coarse. Cardiac auscultation was negative for abnormalities. The abdomen was soft with upper abdominal tenderness most significant on the left, rebound tenderness, and Murphy’s sign equivocal. No abdominal masses or shifting dullness was detected. Questions in Scene One: (1) Which areas should be focused on during the physical exam? (2) What urgent tests need to be arranged initially? (3) What acute abdomen should be considered for a preliminary diagnosis?

Emergency tests included blood count, liver and renal function, lipid profile, amylase levels, ECG, and chest-abdominal CT. Results: WBC 13.2 × 10^9^/L, NEU% 87.5%, RBC 4.16 × 10^9^/L; total bilirubin 46.8 μmol/L, ALT 219 u/L, AST 457 u/L; blood amylase 1690 u/L; CT showed inflammation in both lungs, gallstones, and pancreatic swelling with peripancreatic fluid collection. Slight abnormalities were found in lipid and renal function. Diagnosed with acute pancreatitis and gallstones, the patient was admitted to the emergency surgical ward, receiving anti-inflammatory, enzyme-inhibiting, hepatoprotective, and nutritional treatment, which led to symptom improvement. However, 3 days later, the patient reported increasing right upper abdominal pain, accompanied by cold sweat and dark urine, followed by jaundice and fever up to 39.5 °C. Questions in Scene Two: (1) Why did the abdominal pain initially improve but then worsen? (2) What conditions should be considered when jaundice and dark urine appear during the illness? (3) What diagnostic and therapeutic steps are needed next?

The attending physician noted symptoms of right upper abdominal pain, fever, and jaundice, and an emergency hepatobiliary ultrasound indicated an enlarged common bile duct with mid-distal stones. Blood count re-evaluation: WBC 20.6 × 10^9^/L, NEU% 91.4%, RBC 3.97 × 10^9^/L, suggesting gallstones migrating to the common bile duct causing cholangitis. Despite changing antibiotics to cefoperazone-sulbactam, the patient’s fever persisted, with decreased blood pressure and apathetic expression. An emergency cholecystectomy, bile duct exploration, stone removal, and T-tube drainage were performed, leading to patient improvement and discharge. Questions in Scene Three: (1) What acute abdomen should cholangitis be differentiated from? (2) What are the surgical principles for treating severe acute cholangitis? (3) What should be observed during biliary pancreatitis postoperative recovery? During discussion, interns analyzed the case using mind mapping and completed clinical scenario simulations.

### 3.2 Comparison of pre- and post-teaching performance in both groups

**FIGURE 3:**
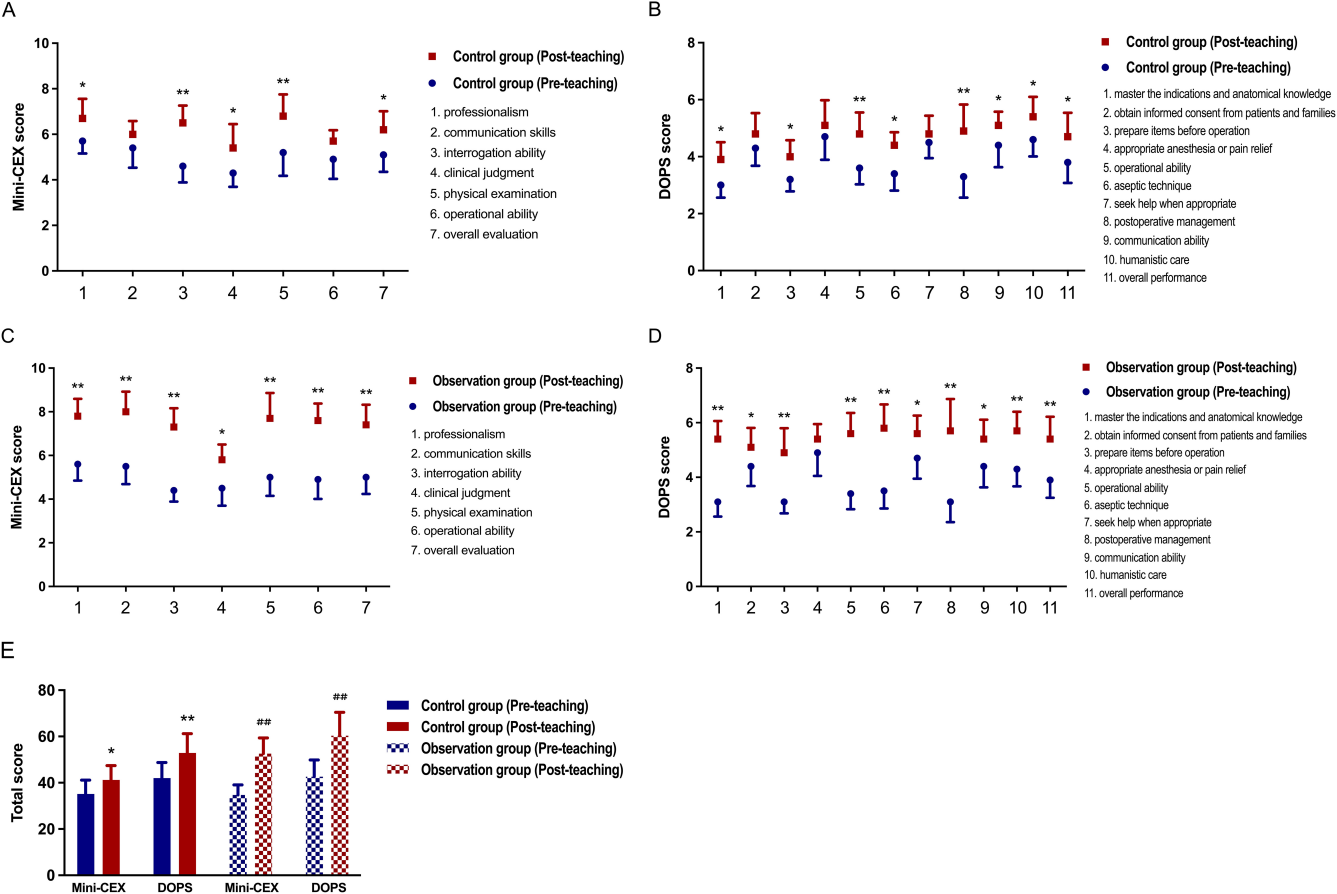
Within-group competency improvement (pre- vs. post-teaching). **(A,B)** Compares individual Mini-CEX and DOPS scores in control group before and after instruction, with *P < 0.05, **P < 0.01 versus pre-teaching control group; **(C,D)** compares individual Mini-CEX and DOPS scores in observation group before and after instruction, with *P < 0.05, **P < 0.01 versus pre-teaching observation group; **(E)** compares comprehensive Mini-CEX and DOPS scores in groups before and after instruction, with *P < 0.05, **P < 0.01 versus pre-teaching control group, ##*P* < 0.01 versus pre-teaching observation group.

Interns in both the control and observation groups were evaluated using Mini-CEX and DOPS assessments before and after participating in departmental teaching research. Changes in scores helped evaluate their proficiency in diagnosing and managing acute abdomen cases, as well as their clinical procedural skills. The results indicate substantial improvement in Mini-CEX and DOPS scores for both groups post-teaching, affirming that both CPBL with mind mapping and traditional lecture-based learning teaching effectively enhance job competency in interns, as demonstrated in Figure 3.

### 3.3 Comparative analysis of teaching effects between groups

**FIGURE 4:**
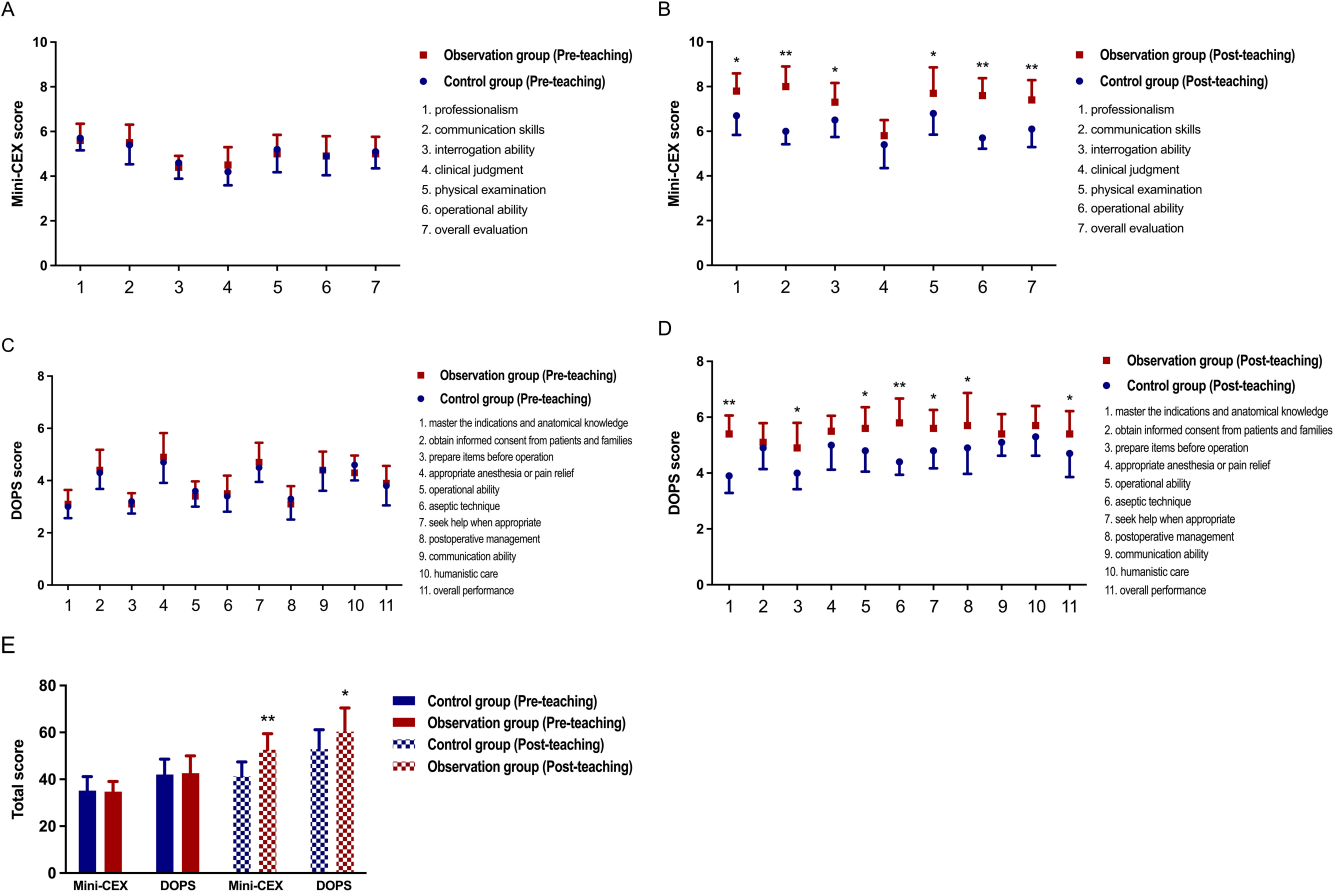
Between-group comparison of teaching effectiveness (control vs. observation). **(A,B)** Compares individual Mini-CEX scores between groups before and after instruction, with *P < 0.05, **P < 0.01 versus post-teaching control group; **(C,D)** compares individual DOPS scores between groups before and after instruction, with *P < 0.05, **P < 0.01 versus post-teaching control group; **(E)** compares comprehensive Mini-CEX and DOPS scores between groups before and after instruction, with *P < 0.05, **P < 0.01 versus post-teaching control group.

Interns from both groups underwent assessments of diagnostic and procedural skills before and after participating in departmental teaching research. By analyzing Mini-CEX and DOPS scores, differences in the teaching effectiveness of various training models were determined. Initial assessments showed no significant differences between the two groups, but post-teaching results indicate higher scores for the observation group, suggesting that CPBL with mind mapping better improves job competency in interns, as seen in Figure 4.

### 3.4 Evaluation of teaching adherence and satisfaction between both groups

Effective teaching outcomes under different training modalities closely relate to the degree of adherence. Evaluations considered standardized practice, proactive learning, and compliance with assessment, categorizing adherence levels as complete, basic, partial, or none, with the former two indicating better adherence. Results showed good adherence in both groups (Table 2).

**TABLE 2:** Comparison of the members of the two groups in teaching adherence [*n* (%)].

Group	Number of cases	Full compliance	Basic compliance	Partial compliance	Non-compliance	Adherence
Observation group	144	86	35	17	6	121 (84.0%)
Control group	72	44	19	6	3	63 (87.5%)
*x* ^2^						0.862
*P*						0.478

Pre-exit satisfaction assessments for intern doctors used a custom survey focusing on teaching model acceptance, learning motivation, teamwork enhancement, clinical reasoning improvement, knowledge comprehension, and overall satisfaction. Survey results showed significantly higher satisfaction scores for the observation group, suggesting a preference for CPBL with mind mapping aimed at improving job competency (Table 3).

**TABLE 3:** Comparison of the members of the two groups in teaching satisfaction.

Group	Number of cases	Recognizing teaching mode	Stimulating learning interest	Enhancing teamwork	Improving clinical thinking	Deepening knowledge understanding	Overall satisfaction	Overall scores
Observation group	144	9.23 ± 1.42	8.91 ± 1.03	8.72 ± 0.96	9.05 ± 1.66	8.56 ± 0.90	9.30 ± 1.45	53.87 ± 7.42
Control group	72	8.35 ± 1.19	7.50 ± 0.85	6.54 ± 1.02	7.87 ± 1.23	7.61 ± 1.26	7.38 ± 1.18	45.23 ± 6.75
*t*		2.826	4.087	5.529	3.614	3.242	5.160	4.728
*P*		0.024	0.003	<0.001	0.007	0.015	<0.001	<0.001

## 4 Discussion

The 21st century marks a pivotal period of rapid advancement and continual innovation in higher medical education, with educators reforming theoretical and practical curricula to develop competent medical professionals to meet societal needs (21). Emergency surgery, as an emerging clinical specialty, has gained considerable attention and is seen as a reflection of a hospital’s comprehensive surgical capabilities. Through effective clinical teaching, interns’ competency in managing acute abdomen cases can be enhanced (22). Pressures of preparing for postgraduate entrance examination, employment concerns, lack of clinical experience, and insufficient communication with instructors often diminish interns’ initiative in clinical learning (23, 24). Thus, improving teaching methods to foster enthusiasm in emergency surgery can enhance students’ clinical reasoning, surgical skills, and physician-patient communication abilities.

By participating in diverse instructional training activities, such as organ/system integration, PBL, scenario simulation, and MOOCs, and combining with the diagnosis and treatment characteristics of acute abdomen patients in the department, this study has decided to implement CPBL with mind mapping for enhancing job competency among medical interns (25–27). Interns engage in analyzing and solving real acute abdomen cases, with teamwork deepening their understanding of theoretical knowledge while fostering clinical reasoning and procedural skills. Mind mapping supplements CPBL by utilizing markers, lines, and images to document thought processes, focusing on clinical symptoms of acute abdomen cases, seamlessly integrating various stages of disease progression and management. Clinical scenario simulations further strengthen interns’ skills in information synthesis and problem-solving (28).

A fair and rational assessment framework ensures objective evaluation of internship teaching efficacy, in which Mini-CEX and DOPS can facilitate simultaneous assessment and feedback of clinical competencies, and prompt interns to reflect upon and improve their learning (29, 30). Post-teaching, both the groups in this research demonstrated significant improvement in Mini-CEX and DOPS scores, with the observation group showing greater progress compared to controls, affirming that CPBL with mind mapping surpasses traditional lecture-based learning teaching in boosting the job competency of interns in acute abdomen management. Both groups exhibited adaptability and cooperation in their instructional activities, and completion of satisfaction surveys post-rotation indicated higher acceptance and interest in CPBL with mind mapping. Therefore, this clinical teaching model is deemed to possess superior applicability. In addition, we are preparing to collaborate with departments such as thoracic surgery, gynecology, neurology and pediatrics to further assess whether this teaching method is generally effective in various medical specialties. Of course, to avoid situations like Hawthorne and novelty effects, where some participants temporarily improve their performance due to new teaching method or to gain more attention, we are also actively promoting a more reasonable training and assessment system (31).

## 5 Conclusion

In conclusion, acute abdomen cases admitted to emergency surgery often present severe conditions with varied clinical symptoms, making differential diagnosis challenging. Therefore, fostering medical students’ professionalism, correct diagnostic and treatment approaches, swift adaptability, and precise technical skills is crucial. Implementing CPBL with mind mapping in acute abdomen clinical teaching, and utilizing appropriate quantitative assessment tools to analyze educational outcomes, aims to shift from teacher-centered to student-focused learning models (32). This initiative not only enhances interns’ job competency but also stimulates learning enthusiasm and can be further considered and implemented in internship training at hospitals nationwide.

## Data Availability

The raw data supporting the conclusions of this article will be made available by the authors, without undue reservation.

## References

[1] Bedross A Siraw B Alkhidir A Zaher E Patel P Kumar A The impact of an intern’s clinical guidebook on easing the transition of new interns into the United States healthcare system. *Cureus.* (2024) 16:e54874. 10.7759/cureus.54874 38533177 PMC10964215

[2] Ganjitsuda K Tagawa M Tomihara K Saiki T Kikukawa M Takamura A Long-term clinical clerkship improves medical students’ attitudes toward team collaboration. *Int J Med Educ.* (2022) 13:274–86. 10.5116/ijme.633f.e97a 36327444 PMC9911282

[3] Ruiz-Manzanera J Almela-Baeza J Aliaga A Ádanez G Alconchel F Rodríguez J Validation of checklists and evaluation of clinical skills in cases of abdominal pain with simulation in formative, objective, structured clinical examination with audiovisual content in third-year medical students’ surgical clerkship. *J Surg Educ.* (2024) 81:1756–63. 10.1016/j.jsurg.2024.08.016 39305605

[4] Li Y Gao C Zhu X Zhu J Ding Z Han Z. Application of SPARK teaching in acute abdomen radiography teaching for undergraduate medical students. *BMC Med Educ.* (2022) 22:881. 10.1186/s12909-022-03957-9 36536422 PMC9762863

[5] Stumbar S Khamisani N Bhoite P Uchiyama E Stevens M Saunders J Abdominal pain in a gender-diverse patient: a standardized patient case for second-year medical students. *Cureus.* (2025) 17:e77873. 10.7759/cureus.77873 39991381 PMC11847163

[6] Yew K George M Allred H. Acute abdominal pain in adults: evaluation and diagnosis. *Am Fam Physician.* (2023) 107:585–96. 37327158

[7] Frija-Gruman N Steinert Y Macdonald M Sun N. Learning through teaching: how physicians learn medicine in authentic clinical contexts. *Acad Med.* (2025) 100:306–12. 10.1097/ACM.0000000000005662 38363796

[8] Dehmoobad Sharifabadi A Clarkin C Doja A. Perceptions of competency-based medical education from medical student discussion forums. *Med Educ.* (2019) 53:666–76. 10.1111/medu.13803 30690769

[9] Yan J Wen Y Liu X Deng M Ye B Li T The effectiveness of problem-based learning and case-based learning teaching methods in clinical practical teaching in TACE treatment for hepatocellular carcinoma in China: a bayesian network meta-analysis. *BMC Med Educ.* (2024) 24:665. 10.1186/s12909-024-05615-8 38886707 PMC11184776

[10] García-Ponce ÁL Martínez-Poveda B Blanco-López Á Quesada AR Suárez F Alonso-Carrión FJ A problem-/case-based learning approach as an useful tool for studying glycogen metabolism and its regulation. *Biochem Mol Biol Educ.* (2021) 49:236–41. 10.1002/bmb.21449 32897596

[11] Peng Y Yang L Qi A Zhang L Xiong R Chen G. Simulation-based learning combined with case and problem-based learning in the clinical education of joint surgery. *J Surg Educ.* (2023) 80:892–9. 10.1016/j.jsurg.2023.03.001 37032261

[12] Mohammed Z Ba H Nasim L Reid E. Supporting Muslim undergraduate medical students through medical school: lessons from a novel, student-led case-based learning intervention. *Front Med.* (2025) 12:1545437. 10.3389/fmed.2025.1545437 40417669 PMC12098333

[13] Xie W Li Y Liu X. Application of problem-based learning and case-based learning in teaching ectopic pregnancy to fifth-year medical students. *BMC Med Educ.* (2024) 24:1346. 10.1186/s12909-024-06327-9 39574098 PMC11583762

[14] Havyer R Nelson D Wingo M Comfere N Halvorsen A McDonald F Addressing the interprofessional collaboration competencies of the association of american medical colleges: a systematic review of assessment instruments in undergraduate medical education. *Acad Med.* (2016) 91:865–88. 10.1097/ACM.0000000000001053 26703415

[15] Hu Z Zhang W Huang M Liu X. Application of directly observed procedural skills in hospital infection training: a randomized controlled trial. *Front Med.* (2025) 12:1509238. 10.3389/fmed.2025.1509238 40432726 PMC12106404

[16] Nunnink L Thompson A Alsaba N Brazil V. Peer-assisted learning in simulation-based medical education: a mixed-methods exploratory study. *BMJ Simul Technol Enhanc Learn.* (2020) 7:366–71. 10.1136/bmjstel-2020-000645 35515740 PMC8936843

[17] Ali S Merdjanoff A Parekh N DiClemente R. Development of an integrated approach to virtual mind-mapping: methodology and applied experiences to enhance qualitative health research. *Qual Health Res.* (2022) 32:571–80. 10.1177/10497323211058161 34847809

[18] Hu Y Xie M. Exploring the benefits of mind mapping in teaching pre-anesthetic evaluation for anesthesia residents. *Asian J Surg.* (2024) 47:5393–4. 10.1016/j.asjsur.2024.06.069 38964965

[19] Sivaraman G Lakshmanan J Alexander A Mahalakshmy T Raja K Sabharisan P Use of Mini-CEX as formative assessment tool in the training of undergraduate medical students in ENT situation analysis and the way forward. *Indian J Otolaryngol Head Neck Surg.* (2024) 76:2698–703. 10.1007/s12070-023-04461-2 38883525 PMC11169284

[20] Luo P Shen J Yu T Zhang X Zheng B Yang J. Formative objective structured clinical examination with immediate feedback improves surgical clerks’ self-confidence and clinical competence. *Med Teach.* (2023) 45:212–8. 10.1080/0142159X.2022.2126755 36151754

[21] Hope D Dewar A Hay C. Is there a replication crisis in medical education research? *Acad Med.* (2021) 96:958–63. 10.1097/ACM.0000000000004063 33735127

[22] Cioffi S Benuzzi L Herbolzheimer M Marrano E Bellio G Kluijfhout W Identifying and addressing mentorship gaps in European trauma and emergency surgical training. Results from the Young European society of trauma and emergency surgery (yESTES) mentorship survey. *Eur J Trauma Emerg Surg.* (2024) 50:2539–49. 10.1007/s00068-024-02610-y 39120653 PMC11599355

[23] Khawaji B Alorabi R Alzahrani R Alhnaidi B Alzahrani R Althobaiti R Medical interns and senior medical students’ perceptions toward clinical teaching. *BMC Med Educ.* (2025) 25:1245. 10.1186/s12909-025-07864-7 40898212 PMC12403378

[24] Sternszus R Slattery N Cruess R Cate O Hamstra S Steinert Y. Contradictions and opportunities: reconciling professional identity formation and competency-based medical education. *Perspect Med Educ.* (2023) 12:507–16. 10.5334/pme.1027 37954041 PMC10637293

[25] Xu Z Liu N Zhang J Wu X Chen J Chang J Application of symptom-based mind mapping combined with PBL teaching method in emergency trauma standardized resident training in MDT model. *Medicine.* (2022) 101:e30822. 10.1097/MD.0000000000030822 36197173 PMC9509091

[26] Sun Q Pang Y Liu X He M Dong J Xie J. Enhancing general surgery clerkships: the application and value of standardized patient-based situational simulation teaching. *Cureus.* (2024) 16:e60845. 10.7759/cureus.60845 38910777 PMC11191846

[27] Huang Z Yang J Wang H Chen B Zheng D Chen H. Integration of massive open online Course (MOOC) in ophthalmic skills training for medical students: outcomes and perspectives. *Asia Pac J Ophthalmol.* (2022) 11:543–8. 10.1097/APO.0000000000000548 36417679

[28] Anton N Rendina M Hennings J Stambro R Stanton-Maxey K Stefanidis D. Association of medical students’ stress and coping skills with simulation performance. *Simul Healthc.* (2021) 16:327–33. 10.1097/SIH.0000000000000511 33086369

[29] Li X. Application of PBL-CBL and Mini-CEX methods in the standardized training of residents in nephrology department: a prospective study. *Pak J Med Sci.* (2024) 40:2046–51. 10.12669/pjms.40.9.9434 39416615 PMC11476161

[30] Rela M Price T. Review of the validity of DOPS as an assessment tool for the procedural skills of surgical trainees. *Ann R Coll Surg Engl.* (2023) 105:599–606. 10.1308/rcsann.2022.0052 36374304 PMC10471438

[31] Paradis E Sutkin G. Beyond a good story: from hawthorne effect to reactivity in health professions education research. *Med Educ.* (2017) 51:31–9. 10.1111/medu.13122 27580703

[32] Huang C Xiao Y Xu D Wong I Patil N Chen J Developing a student-centred curriculum: insights from anaesthetic placement experiences for co-designing in China’s greater bay area. *Adv Med Educ Pract.* (2025) 16:1695–704. 10.2147/AMEP.S530349 40989088 PMC12452981

